# Genetic characterization of dengue virus serotype 1 circulating in Reunion Island, 2019–2021, and the Seychelles, 2015–2016

**DOI:** 10.1186/s12879-023-08125-y

**Published:** 2023-05-05

**Authors:** Sarah Hafsia, Tatiana Barbar, David A Wilkinson, Célestine Atyame, Leon Biscornet, Jastin Bibi, Meggy Louange, Jude Gedeon, Olga De Santis, Antoine Flahault, André Cabie, Antoine Bertolotti, Patrick Mavingui

**Affiliations:** 1 UMR Processus Infectieux en Milieu Insulaire Tropical (PIMIT), Université de La Réunion, CNRS 9192, INSERM U1187, IRD 249, Plateforme CYROI, Sainte Clotilde, La Réunion, France; 2 Public Health Authority, Ministry of Health, Victoria, Seychelles; 3 Disease Surveillance and Response Unit, Epidemiology and Statistics Section, Public Health Authority, Ministry of Health, Victoria, Seychelles; 4 Institute of global health, Faculty of Medicine, University of Geneva, Geneva, Switzerland; 5 CHU de Martinique, service de maladies infectieuses et tropicales, INSERM, CHU de Martinique, PCCEI, Univ Montpellier, Univ Antilles, INSERM, EFS, CIC1424 Fort-de-France, Montpellier, France; 6 Service des Maladies Infectieuses - Dermatologie, CHU Réunion, INSERM CIC1410, Saint Pierre, Saint Pierre, La Réunion, France

**Keywords:** Dengue, Outbreaks, Genetics, Genomes, Reunion Island, Seychelles

## Abstract

**Background:**

An unprecedent increase in the number of cases and deaths reported from dengue virus (DENV) infection has occurred in the southwestern Indian ocean in recent years. From 2017 to mid-2021 more than 70,000 confirmed dengue cases were reported in Reunion Island, and 1967 cases were recorded in the Seychelles from 2015 to 2016. Both these outbreaks displayed similar trends, with the initial circulation of DENV-2 which was replaced by DENV-1. Here, we aim to determine the origin of the DENV-1 epidemic strains and to explore their genetic characteristics along the uninterrupted circulation, particularly in Reunion.

**Methods:**

Nucleic acids were extracted from blood samples collected from dengue positive patients; DENV-1 was identified by RT-qPCR. Positive samples were used to infect VERO cells. Genome sequences were obtained from either blood samples or infected-cell supernatants through a combination of both Illumina or MinION technologies.

**Results:**

Phylogenetic analyses of partial or whole genome sequences revealed that all DENV-1 sequences from Reunion formed a monophyletic cluster that belonged to genotype I and were closely related to one isolate from Sri Lanka (OL752439.1, 2020). Sequences from the Seychelles belonged to the same major phylogenetic branch of genotype V, but fell into two paraphyletic clusters, with greatest similarity for one cluster to 2016–2017 isolate from Bangladesh, Singapore and China, and for the other cluster to ancestral isolates from Singapore, dating back to 2012. Compared to publicly available DENV-1 genotype I sequences, fifteen non-synonymous mutations were identified in the Reunion strains, including one in the capsid and the others in nonstructural proteins (NS) (three in NS1, two in NS2B, one in NS3, one in NS4B, and seven in NS5).

**Conclusion:**

In contrast to what was seen in previous outbreaks, recent DENV-1 outbreaks in Reunion and the Seychelles were caused by distinct genotypes, all likely originating from Asia where dengue is (hyper)endemic in many countries. Epidemic DENV-1 strains from Reunion harbored specific non-synonymous mutations whose biological significance needs to be further investigated.

**Supplementary Information:**

The online version contains supplementary material available at 10.1186/s12879-023-08125-y.

## Background

Dengue virus (DENV) is the most widespread mosquito-borne flavivirus worldwide. More than 390 million people in over 129 countries are exposed to dengue virus infections with an estimated 20,000 deaths every year [1, 2]. DENV is transmitted through the bite of infected female mosquitoes belonging to the *Aedes* genus; the species *Aedes aegypti* being a primary vector while *Aedes albopictus* plays a secondary role [3]. The DENV genome consists of a positive-sense single-stranded linear RNA of approximately 11 kilobases. Four DENV serotypes (DENV-1 to DENV-4) are currently known. Molecular analyses have highlighted the diversity of DENV genotypes belonging to each of the four serotypes [4, 5].

The southwestern Indian ocean islands have a history of periodic outbreaks of DENV, but Reunion Island has been an exception in recent years. Since an outbreak of the virus in 2017, there has been continuous circulation of DENV on the island with over 70,000 confirmed cases between 2017 and 2022 (Table 1). This continuous presence of the virus on the island may suggest that dengue has become endemic [6].

**Table 1:** Circulation of DENV from 2017 to mid-2022 in Reunion

Year*	Number of confirmed cases	Number of serotyped cases (TotS)	DENV-1/TotS	DENV-2/TotS	DENV-3/TotS
2017	94	94	0	1	0
2018	6,759	950	0	1	0
2019	18,217	883	0.118	0.880	0.002
2020	16,414	838	0.85	0.12	0.03
2021	29,655	978	1	0	0
2022	1,129	329	1	0	0

*Data were obtained from [6] and Santé Publique France [7, 8].

At the beginning of the epidemic wave, DENV-2 was the first serotype detected on the island [9, 10]. At the end 2019, among the 25,000 confirmed cases and 20 deaths, DENV-2 still represents the major serotype, but a few cases of DENV-1 and DENV-3 were also reported [11]. During the course of the epidemic, DENV-1 was also observed to co-circulate with DENV-2, and rapidly became the dominant serotype. DENV-1 was the only identified serotype among the 29,577 confirmed cases by week 35 in 2021 [12]. A similar trend was also observed in the Seychelles where an outbreak of DENV-2 started in 2015, being replaced by DENV-1 in 2016 (MOH Seychelles). Interestingly, the DENV-2 strains that circulated at the beginning of these outbreaks are 99.8% similar to each other and 93% similar to a 2013 strain from Singapore, indicating a single introduction of DENV-2, presumably from Asia [10]. As DENV-1 subsequently succeeded DENV-2 in these two islands, it would be interesting to see whether the DENV-1 outbreaks were also initiated by a single introduction event.

In 2021, DENV-1 circulation in Reunion coincided with a significant increase of severe cases and hospitalizations when compared to 2018 and 2019 [12]. For instance, several severe ophthalmological cases related to DENV-1 infection were reported, notably in 2020 and 2021 [12]. Moreover, while severe forms and deaths were most frequently observed in older patients with comorbidities such as diabetes and hypertension, increasing numbers of DENV1-induced mortalities were observed in younger patients (including children) without comorbidities [12]. Although such an increase in severity was observed for the first time in Reunion, these symptoms were already described in Asia where dengue is endemic [13, 14]. To investigate whether the current epidemic DENV-1 strains from Reunion and the Seychelles were similar to each other and to those known in Asia, we performed whole-genome sequencing and phylogenic analyses to trace their origin and explore some genetic features.

## Methods

### Origin of samples

Biological samples from Reunion Island were originated from two different collections. The CARBO collection consisted of a prospective cohort of patients with arbovirus infections, registered on clinicaltrials.gov (NCT01099852). The CARBO was started by the University Hospital of La Martinique in 2010 and extended to include patients from Reunion in 2018. The other collection, DEMARE, was established as part of an epidemiological cross-sectional study conducted in 2019–2020. Both collections were approved by The French Committee for the Protection of Individuals. Written consent was obtained from all participants. Blood samples from the Seychelles were collected by the Ministry of health of the Seychelles during outbreaks between January 2015 and December 2016.

Bloods were collected from serologically confirmed patients presenting one or several dengue symptoms including fever, myalgia, headache, asthenia and thrombocytopenia and without recent travel history. To increase the probability of detecting the presence of viral RNA, the samples used in the study were from patients included in the cohort within the first seven days of the onset of symptoms. Collections were performed by accredited professionals in 10 mL EDTA tubes. Within the two hours after collection, samples were centrifuged at 2,000 g for 10 min at 20 °C. Supernatants were transferred into new tubes, homogenized by inversion, aliquoted into new 2 mL tubes, labeled, then frozen and stored at -80 °C until used.

### Viral RNA extraction and amplification

To ascertain the presence and the amount of DENV-1 RNA in the selected samples, a reverse transcription quantitative real-time PCR (RT-qPCR) was performed. Briefly, total nucleic acids were extracted from the serum samples using the QIAamp® Mini Kit purification according to the manufacturer’s recommendation (QIAGEN). For RT-qPCR, we used the QIAGEN OneStep kit following the manufacturer’s instructions (QIAGEN). A mixed solution was prepared with RNA template (5 µl), a TaqMan probe (FAM-ACACCTCAAGCTAA-TAMRA) and primers (Forward 5’-GAACATGGRACAAYTGCAACYAT-3’; Reverse 5’-CCGTAGTCDGTCAGCTGTATTTC-3’) specific to DENV-1. The thermocycler program consisted of a retrotranscription step of 45 min at 45 °C, denaturation for 5 min at 95 °C followed by 40 cycles of amplification (72 °C for 5 s and 56 °C for 60 s). Viral RNA copy number was estimated against a standard curve following the methodology published by the HAS (Haute Autorité de Santé, France). Plasmids containing targeted DENV-1 were synthesized by GeneCust (France) and used as the standard curve at concentrations of 10^1^ to 10^8^ RNA copy per µl.

The E gene was amplified to confirm RT-qPCR-positive samples. cDNA was synthesized from extracted RNA using the ProtoScript® II Reverse Transcriptase Kit with random primers following the manufacturer’s instructions (New England BioLab, USA) and amplified using a nested PCR protocol, as previously described [9]. Briefly, we designed degenerated primers targeting a fragment (~ 700–800 bp) of the E gene encoding the envelope protein. The first round of amplification reaction was performed with primers DNV1-E-F1 (CAC TGG TGG AAG AAC AAG ACG C) and DNV1-E-R2 (CMA CDG AYG TGA ACA CYC CTC C) generating an approximately 1100-bp fragment. The second round used the primers DNV1-E-F2 (ACG GAG CTC TYA CAT TGG ACT G) DNV1-E-R2 (CMA CDG AYG TGA ACA CYC CTC C) and generated a 750-bp fragment. PCR amplification was performed on a PCR System 2700 Thermocycler (ABI Applied Bio-system ™). Amplification programs were as follows: 94 °C for 2 min; 3 cycles of 95 °C for 5 s, 60 °C for 30 s, 72 °C for 30 s; 3 cycles of 95 °C for 5 s, 55 °C for 30 s, 72 °C for 30 s; and 28 cycles of 95 °C for 5 s, 50 °C for 30 s, 72 °C for 1 min 30 s; 72 °C for 7 min. Amplified products were checked first on electrophoresis gel (Additional file) then samples showing amplicons of expected sizes were Sanger sequenced (Genoscreen, Lille, France).

### Virus isolation

Viral isolation assays were conducted by inoculating the DENV-1 PCR-positive sera from viremic patients onto Vero E6 cell monolayers. Cultures were checked every day. When cytopathic effects were observed, supernatants (passage 1) were collected by centrifugation and stored at -80 °C. The presence of viral RNA was confirmed by RT-qPCR, as above, and by titration of viral infectious particles using plaque-forming unit assay [10].

### Sequencing and genomic analysis

Genomic data were generated using either Oxford Nanopore Technologies (MinION) sequencing based on the amplicon tiling protocol, or Illumina shotgun sequencing, or by combining data from both sequencing platforms [15].

For Illumina sequencing, libraries were generated from 10 ng of cDNA using the Celero™ PCR Workflow with Enzymatic fragmentation (DNA-Seq) following the manufacturer’s instructions. Sequencing was performed on the MiSeq platform, with 1 * 170 bp single-end reads. Demultiplexed sequences were provided by the sequencing company (Biofidal, Lyon, France). Sequences were quality trimmed and adapters removed using Trimmomatic v0.39 [16]. Trimmed reads were mapped to reference sequence NC_001477.1 using bowtie2 [17]. Geneious v9.1.8 [18] was used to inspect and curate mapped sequence data.

For Oxford Nanopore Technology’s MinION sequencing, an amplicon tiling protocol was used in conjunction with DENV1 primers from the Oxford Centre for Arbovirus Discovery, Diagnostics, Genomics and Epidemiology (https://www.caddecentre.org/protocols/). Briefly, cDNA was amplified in two independent PCR reactions. PCR products were pooled, dA-tailed using the NEBNext® Ultra™ II End Repair/dA-Tailing Module then barcoded using the Nanopore Native Barcoding Expansion kit (EXP-NBD104). Barcoded amplicons were then purified using a 0.4x volume of AMPure-XP SPRI beads, washing the beads with an excess volume of Nanopore’s small fragment buffer (SFB) to ensure that un-ligated barcode molecules were removed. Purified amplicons were then pooled in equimolar proportions before adapter ligation and sequencing, following the manufacturer’s instructions. The sequencing run was left for 24 h and stopped when predicted coverage exceeded 1000-fold for each genome. Base-calling and demultiplexing were performed using guppy (v4.0.11). Base-calling used default parameters in accurate mode, and demultiplexing was performed using the “require_barcodes_both_ends” parameter to minimize sample crosstalk. Reads were then assembled using Medaka v1.0.3 (https://nanoporetech.github.io/medaka/) mimicking the ARTIC network bioinformatics standard operating procedure for SARS-CoV-2 sequencing (https://artic.network/ncov-2019/ncov2019-bioinformatics-sop.html), subsampling amplicon coverage to 400x and using genome accession NC_001477.1 for read mapping. Geneious v9.1.8 was used to inspect and curate mapped sequence data. Consensus base-calling required a minimum of 30-fold coverage.

For samples from the Seychelles, only partial genomic assemblies were obtained by this method, as not all primer pairs produced amplified products. The partial assembly data were thus combined with Sanger sequence data from the envelope region of the genome to produce contiguous consensus sequences that spanned a 4,838 bp region at the 5’ end of the DENV genome. This region corresponded to base positions 156–4,994 of the reference genome NC_001477.1 and contained genetic data from the capsid, membrane glycoprotein, envelope, NS1, NS2a and NS2b regions of the genome.

### Phylogenetic analysis

Global phylogenetic comparisons were carried out using the E-gene, which is typically used for identifying dengue genotypes [5]. The E-gene region was extracted from all reference sequences in the NCBI database using custom scripts. Alignments were generated using MAFFT [19], and the alignment was curated by eye in Geneious v9.1.8. IQTree2 (v2.1.3) was used to identify optimal substitution model parameters and to generate a bootstrapped maximum-likelihood phylogeny with 1000 replicates. Phylogenetic tree representations were generated in R, using the “ggtree” package [20].

## Results

### General characteristics

Among the 30 blood samples from Reunion and 14 from Seychelles collected within the first seven days of the onset of symptoms, only nine (PR1583, PR1615, PR1914, PR4443, PR4453, PR4463, PR4483, PR6594, P0409) of 2019 to 2021 outbreaks from Reunion and four (DS16177, DS16229, DS16232, DS16233) of 2016 outbreak from Seychelles tested positive for DENV-1 by RT-qPCR were further analyzed (Table 2). A higher viral titer was detected for the two samples PR1583 and PR6594 with approximately 10^7^ RNA copies/µL. These two samples were used to infect VERO cells allowing the isolation of DENV-1 strains named RUN1-1583 and RUN1-6594, respectively, isolates which were also further analyzed (Table 2). Sequencing with Illumina generated complete genome sequences for 5 blood samples (PR4443, PR4453, PR4463, PR4483, PR6594) and the isolate RUN1-6594 (one passage) from Reunion. MinION technology allowed sequencing of almost complete genomes for 3 sera (PR1615, PR1914, P0409) and the isolate RUN1-1583 (one passage) from Reunion. The sequence of each isolated strain was identical to that obtained from the corresponding blood sample. Using MinION technology, only partial sequences were obtained from the four Seychelles samples. Upon sequence inspection, mismatches were identified in the DENV1_3, DENV1_11 and DENV1_12 primer pairs which may explain inefficient amplification of DENV1 genotype V. Further optimization of the amplicon tiling protocol would be required to allow efficient amplification and sequencing of all DENV-1 genotypes by this method.

**Table 2:** Biological samples and DENV isolates used in this study

Sample	Origin	Biological material	Sequencing technology	Coverage	Viral isolation	Genbank Accession
RUN1-1583	Reunion, 2019	Supernatant, cDNA	MinION, Illumina	Complete	Yes	ON631277
PR1615	Reunion, 2019	Serum, cDNA	MinION	Complete*	No	ON631275
PR1914	Reunion, 2019	Serum, cDNA	MinION	Complete*	No	ON631274
PR4443	Reunion, 2020	Serum, cDNA	Illumina	Complete	No	ON631283
PR4453	Reunion, 2020	Serum, cDNA	Illumina	Complete	No	ON631282
PR4463	Reunion, 2020	Serum, cDNA	Illumina	Complete	No	ON631281
PR4483	Reunion, 2020	Serum, cDNA	Illumina	Complete	No	ON631280
PR6594	Reunion, 2021	Plasma, cDNA	Illumina	Complete	No	ON631278
RUN1-6594	Reunion, 2021	Supernatant, cDNA	Illumina	Complete	Yes	ON631279
P0409	Reunion, 2020	Plasma, cDNA	MinION, llumina	Complete	No	ON631276
DS16177	Seychelles, 2016	Serum, cDNA	MinION	Incomplete, 71.2%	No	ON631270
DS16229	Seychelles, 2016	Serum, cDNA	MinION	Incomplete, 88.4%	No	ON631271
DS16232	Seychelles, 2016	Serum, cDNA	MinION	Incomplete, 76.9%	No	ON631272
DS16233	Seychelles, 2016	Serum, cDNA	MinION	Incomplete, 76.9%	No	ON631273

*Genome coverage is considered complete for Illumina data if the sequence obtained mapped to more than 98% of the DENV-1 reference sequence (Genbank accession number NC_001477.1). The amplicon tiling protocol for MinION used primer positions that did not amplify 155 bp at the 5’ extremity, nor 575 bp at the 3’ extremity of each genome, thus complete contiguous sequences using MinION map to 93% of the same reference sequence.

### Phylogeny

**Fig. 1:**
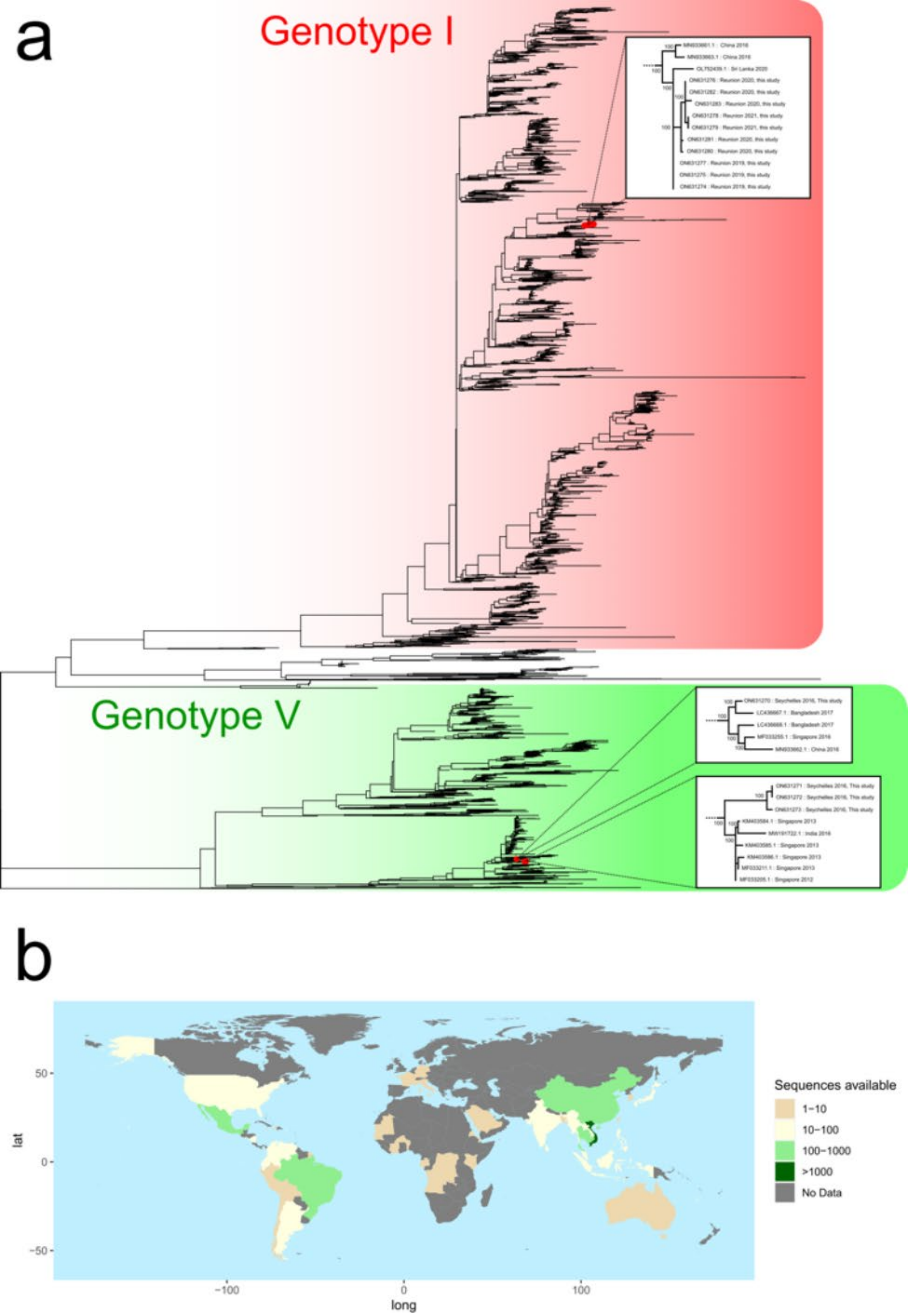
Phylogenetic analyses of DENV. (a) Phylogenetic tree generated using a 4,838-bp region of the DENV genome (positions 156 to 4,994 of NC_001477.1). Inset are zoomed representations of the tree topology and bootstrap values for regions relevant to sequences generated as part of this study. (b) A graphical representation of the country of origin data for sequences included in the phylogenetic analysis

Phylogenetic analyses of the approximately 5000-bp genomic region of 3,891 publicly available sequences showed that all sequences from Reunion Island found in 2019, 2020 and 2021 belonged to genotype I of DENV-1 (Fig. 1a), and possessed nearly identical sequences, forming a monophyletic cluster in the phylogeny. These sequences showed greatest similarity (99.5% identity) to one isolate from Sri Lanka in 2020 (Accession number OL752439.1) and two sequences from China in 2016 (Accession numbers MN933661 and MN933663). Sequences from the Seychelles belonged to the same major phylogenetic branch of DENV-1 genotype V, but fell into two paraphyletic clusters (Fig. 1a). DS16177 showed greatest similarity (> 99% identity) to sequences from 2016 to 2017 isolated in Bangladesh, Singapore and China, whereas DS16229, DS16232, DS16233 and DS16243 showed greatest similarity to numerous ancestral isolates from Singapore, dating back to 2012. However, interpretations of isolate origins should consider the strong sampling bias that exists in available DENV sequence data (Fig. 1b).

Further extending the phylogenetic comparison to include whole genomic data (Additional file) provided no strong evidence for the introduction of separate lineages in Reunion, as all whole genome sequences showed > 99.8% identity (between 0 and 19 SNPs).

### Major non-synonymous mutations

**Fig. 2:**
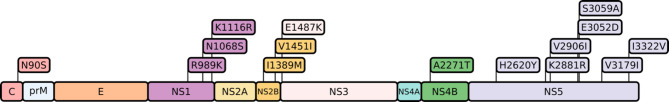
Schematic representation of conserved mutations identified within isolates from Reunion. Position numbers are expressed relative to the first conserved start methionine of all polyprotein sequences belonging to DENV1 as defined in [28]. Mutations are expressed relative to the majority amino acid sequence of DENV1 genotype I sequences from the same dataset. Positions are colored by mature protein: C, Capsid; prM, premembrane; E, envelope; NS, Nonstructrural. A: Alanine; D: Aspartic acid; E: Glutamic acid; H: Histidine; I: Isoleucine; K: Lysine; M: Methionine; N: Asparagine; R: Arginine; S: Serine; T: Threonine; V: Valine; Y: Tyrosine

Fifteen non-synonymous amino acid changes were specifically observed in all Reunion DENV-1 full genome sequences compared with other DENV-1 genotype I sequences publicly available (Fig. 2). Mutations consisted of N90S in the C gene, R989K, N1068S and K1116R in NS1, I1389M, V1451I and E1487K in NS2B, A2271T in NS4B. Seven mutations H2620Y, K2881R, V2906I, E3052D, S3059A, V3179I and I3322V were observed in NS5. As only partial sequences were generated from the Seychelles samples, reconstruction of full polyprotein was not possible, thus precluding the analysis of amino acid changes.

## Discussion

Since the recent dengue outbreak started in late 2017, uninterrupted transmission has occurred in Reunion Island. From 2017 to mid 2022, more than 70,000 confirmed cases were reported [6–8]. Circulation of DENV-2 in 2018 was followed by a major co-circulation of DENV-2 and DENV-1 at the end of 2019 and during 2020, with few cases of DENV-3. In 2021 and 2022, DENV-1 was the only serotype identified in Reunion Island [6]. The Seychelles were also confronted to dengue outbreaks years before (2015–2016) with a similar trend starting with DENV-2 which was replaced by DENV-1 [6].

Using phylogenetic analyses, we showed that the DENV-1 sequences from Reunion were closely related to a 2020 strain circulating in Sri Lanka, whereas those from the Seychelles showed a greatest similarity for isolates from Bangladesh, Singapore and China. This result suggested that the DENV-1 epidemic strains in Reunion and Seychelles were probably imported from Asian countries. We identified a single DENV-1 lineage belonging to genotype I that circulated in Reunion since its emergences at the end of 2019 and subsequently in 2020 and 2021. On the contrary, two lineages of DENV-1 of genotype V occurred in the Seychelles in 2016. It should be noted that these are likely partial descriptions of the total genetic diversity of DENV on the two islands due to the limited sample availability for our study. Even so, our results were distinct to previous observations in the region where similar viral epidemic strains usually occurred in contemporary outbreaks. For instance, between 2003 and 2004 an outbreak caused by DENV-1 serotype started in the Seychelles [21] and then spread in Reunion [22]. The same DENV-1 serotype was involved in an outbreak in Toamasina, eastern Madagascar, two years later in 2006 [23], but a regional link could not be established as no sequence was found. More recently, dengue outbreaks due to DENV-2 occurred in 2016 in the Seychelles and then in 2018 in Reunion, and it was shown to involve the same cosmopolitan sub-lineage I [9, 10]. Moreover, the link of dengue outbreaks between these two islands has been demonstrated by the presence of dengue cases in Reunion imported from the Seychelles whether in 2016 [7] or in 2017 [8].

The presence of two distinct sub-lineages of DENV-1 in the Seychelles suggests at least two introduction events. Conversely, the full-genome phylogenetic analysis (Additional file) strongly suggests that sequenced DENV-1 genotype I strains in Reunion originated from a single introduction event. In these two countries, DENV-1 displaced the previously circulating DENV-2. The displacement phenomenon was also reported among genotypes of the same dengue serotype. For instance, during the DENV-2 outbreaks in several South American countries, the American genotype was replaced by the Asian genotype [24]. In some cases, lineage replacements were associated with an increased clinical severity [24, 25]. In Reunion, the introduction and subsequent dominance of DENV-1 was accompanied by severe cases with peculiar symptoms, notably the maculopathy. Ophthalmic complications associated with dengue, with certain dependence to serotype, have been increasingly described in recent times [13, 26]. The possible link between maculopathy observed in Reunion dengue patients and the emerging lineage DENV-1 genotype I needs to be further investigated.

Moreover, the probable single introduction of the DENV-1 genotype I in Reunion may have been be accompanied by a discrete micro-evolution event since fifteen exclusive non-synonymous mutations were found in different proteins, including capsid, NS1, NS2B, NS3, NS4B, and NS5. Such local micro-evolution events have been observed in Asia, as shown for instance in the Hunan province where several mutations were seen in DENV-2 outbreak (2018) after importation from neighboring areas having higher incidence of dengue [27]. The micro-evolution event in either structural or non-structural proteins can be neutral or under positive or negative selection with an impact in disease epidemiology [27, 28]. For instance, mutations in NS1 have been shown to influence production and secretion with impact on NS1 ELISA-based dengue detection in clinical samples [29]. In addition, it was shown that mutations involving changes from basic to acidic residues or vice versa tend to affect NS1 surface expression and secretion patterns of flavivirus with impact in host immunity [29, 30]. Here, mutations were observed also in both NS2B and NS5, two proteins that play a pivotal role in the activity of NS3 protease, the latter being also a therapeutic target against flaviviruses [31]. Strikingly, when studying mutations in NS2B of the flavivirus Zika, researchers found one particular mutation able to enhance transmission potential and to confer escape from pre-existing DENV immunity [32]. When analyzing several natural dengue variants from dengue severe patients, from mild to fatal cases, a link was established between mutations in NS5 and the virulent DENV phenotypes [33]. Whether the mutations found in DENV-1 epidemic strain played a role in dengue severe cases observed in Reunion needs to be further investigated. Particular attention should be paid in the three mutations (R989K, N1068S and K1116R) located in NS1, since expression of this nonstructural glycoprotein has been linked to dengue disease severity [14, 34, 35]. In addition, as NS1 antigen is a marker for routine diagnosis in rapid detection of dengue virus infection [36], the impact of these mutations on the efficiency of the tests may require further control.

## Conclusion

The southwestern Indian ocean region is usually subject to dengue outbreaks with co-occurrence of a given dengue serotype alternating intense circulation and inter-epidemic periods. In contrast to what was observed previously, uninterrupted dengue circulation is occurring and we showed that recent DENV-1 outbreaks in Reunion and the Seychelles were caused by distinct genotypes, all probably originating from Asia. Strikingly, the unique DENV-1 genotype I lineage circulating during three consecutive years in Reunion harbored non-synonymous mutations whose biological significance need to be further investigated.

## Electronic supplementary material

Below is the link to the electronic supplementary material.

Supplementary Material 1

Supplementary Material 2

Supplementary Material 3

Supplementary Material 4

## Data Availability

The datasets generated and analysed during the current study are available in the Genbank repository under the accession numbers ON631270, ON631271, ON631272, ON631273, ON631274, ON631275, ON631276, ON631277, ON631278, ON631279, ON631280, ON631281, ON631282, ON631283. Data from the CARBO study are not publicly available to protect patient privacy. They can be requested at the Department of research and innovation, Martinique University Hospital 97261 Fort-de-France.

## Abbreviations

DENV: Dengue virus
DENV-1: Dengue virus serotype 1
DENV-2: Dengue virus serotype 2
DENV-3: Dengue virus serotype 3
NS: Nonstructural protein
CARBO: Cohorte arboviruses
DEMARE: Observational cross-sectional epidemiological study conducted in Madagascar and La Réunion
