# Supplementary figures and images for: Genetic characterization of dengue virus serotype 1 circulating in Reunion Island, 2019–2021, and the Seychelles, 2015–2016
Source: BMC Infect Dis. 2023 May 5;23:294. doi: 10.1186/s12879-023-08125-y (PMC10161969; doi:10.1186/s12879-023-08125-y)

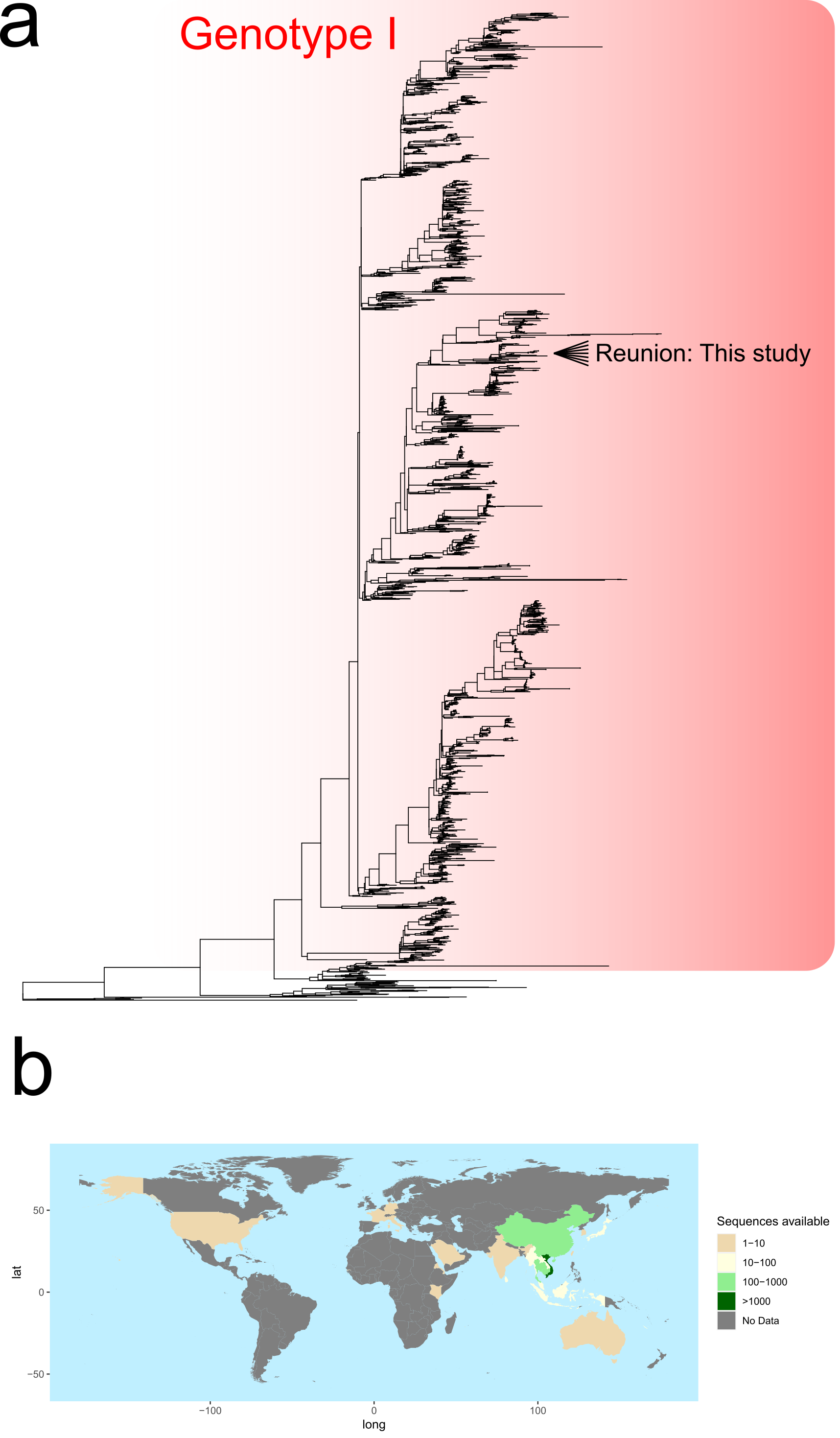

Supplement: Supplementary file 1 — Supplementary Material 1 [file 12879_2023_8125_MOESM1_ESM.png]
