# Supplementary material for: Genetic characterization of dengue virus serotype 1 circulating in Reunion Island, 2019–2021, and the Seychelles, 2015–2016
Source: BMC Infect Dis. 2023 May 5;23:294. doi: 10.1186/s12879-023-08125-y (PMC10161969; doi:10.1186/s12879-023-08125-y)
Supplement: Supplementary file 2 — Supplementary Material 2 [file 12879_2023_8125_MOESM2_ESM.docx]

**Supplementary file legend** : Phylogenomic analyses of DENV. a) Phylogenetic tree using genome sequences generated using Illumina or a combination of Illumina and MinION sequencing technologies. Genome sizes were between 10,516 bp and 10,708 bp in length, covering 98 % - 99 % of the DENV-1 reference genome NC_001477.1. Sequences generated using exclusively the amplicon tiling protocol and MinION sequencing technology lacked 155 bp of data at the 5' extremity and 575 bp at the 3' extremity, with final sequence lengths of 10,004 bp and 10,005 bp, representing approximately 93 % coverage of the reference genome NC_001477.1. b) A graphical representation of the country of origin data for sequences included in the phylogenetic analysis.
