# Supplementary material for: Genetic characterization of dengue virus serotype 1 circulating in Reunion Island, 2019–2021, and the Seychelles, 2015–2016
Source: BMC Infect Dis. 2023 May 5;23:294. doi: 10.1186/s12879-023-08125-y (PMC10161969; doi:10.1186/s12879-023-08125-y)
Supplement: Supplementary file 3 — Supplementary Material 3 [file 12879_2023_8125_MOESM3_ESM.pdf]

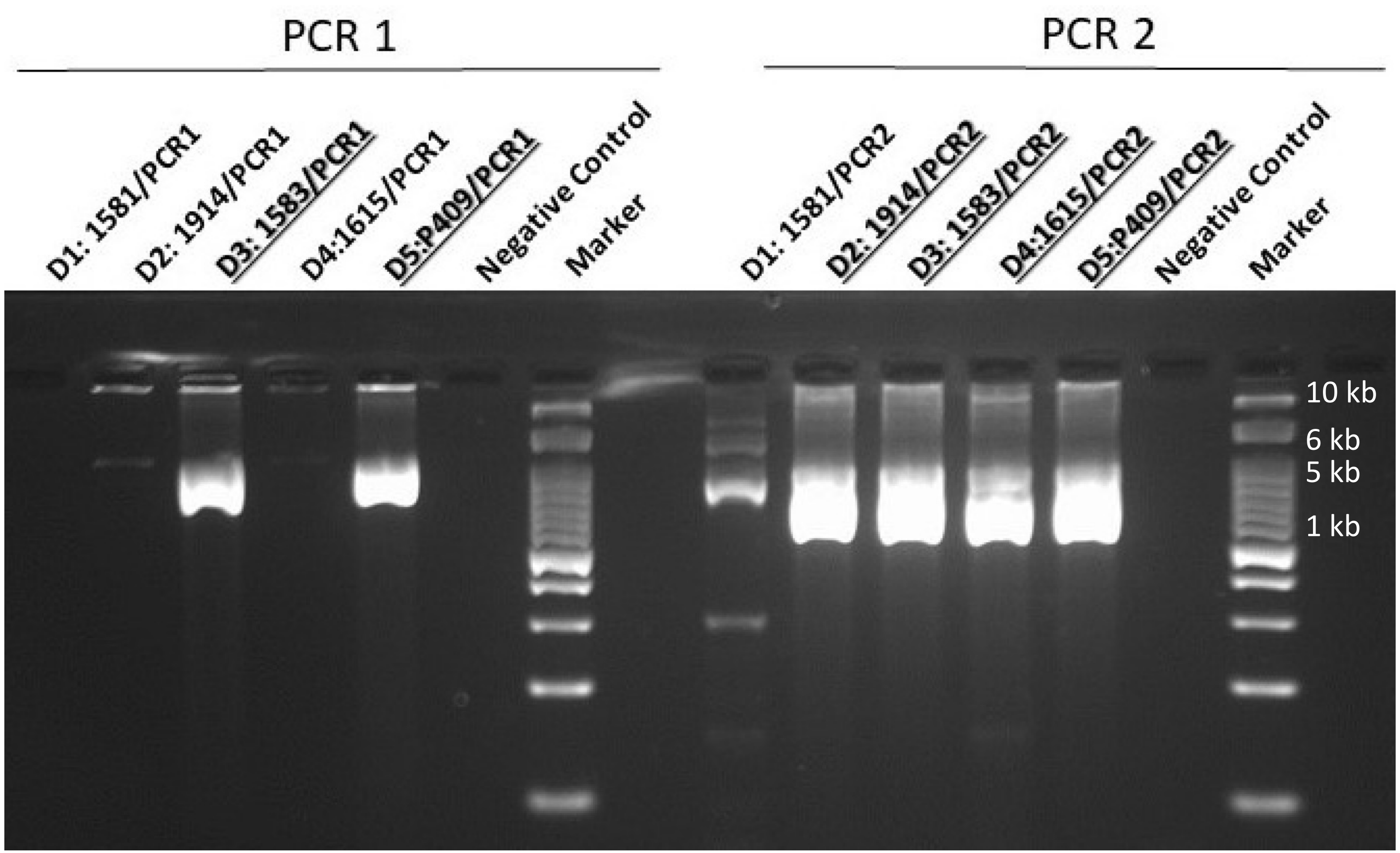
PCR 1
PCR 2
D1: 1581/PCR1
D2: 1914/PCR1
D3: 1583/PCR1
D4:1615/PCR1
D5:P409/PCR1
Negative Control
Marker
D1: 1581/PCR2
D2: 1914/PCR2
D3: 1583/PCR2
D4:1615/PCR2
D5:P409/PCR2
Negative Control
Marker
10 kb
6 kb
5 kb
1 kb
