# Supplementary material for: Genetic characterization of dengue virus serotype 1 circulating in Reunion Island, 2019–2021, and the Seychelles, 2015–2016
Source: BMC Infect Dis. 2023 May 5;23:294. doi: 10.1186/s12879-023-08125-y (PMC10161969; doi:10.1186/s12879-023-08125-y)
Supplement: Supplementary file 4 — Supplementary Material 4 [file 12879_2023_8125_MOESM4_ESM.docx]

Figure: Electrophoresis gel of DENV-1 E gene fragment with example of positive (PR1914, PR1583, PR1615 and P04019) and negative (PR1581) samples collected from patients of Reunion.

PCR 1- Gel 1: Amplicons of the first round of amplification

PCR 2- Gel 2: Amplicons of the second round of amplification

Negative control: water as a template

Marker: 1 kb DNA ladder
